# Bacterial Causes of Empyema in Children, Australia, 2007–2009

**DOI:** 10.3201/eid1710.101825

**Published:** 2011-10

**Authors:** Roxanne E. Strachan, Anita Cornelius, Gwendolyn L. Gilbert, Tanya Gulliver, Andrew Martin, Tim McDonald, Gillian M. Nixon, Rob Roseby, Sarath Ranganathan, Hiran Selvadurai, Greg Smith, Manuel Soto-Martinez, Sadasivam Suresh, Laurel Teoh, Kiran Thapa, Claire E. Wainwright, Adam Jaffé, on behalf of the Australian Research Network in Empyema

**Affiliations:** Author affiliations: Sydney Children’s Hospital, Randwick, New South Wales, Australia (R.E. Strachan, A. Jaffé);; Royal Hobart Hospital, Hobart, Tasmania, Australia (A. Cornelius);; Centre for Infectious Diseases and Microbiology, Westmead, New South Wales, Australia (G.L. Gilbert, K. Thapa);; John Hunter Hospital, Newcastle, New South Wales, Australia (T. Gulliver);; Princess Margaret Hospital for Children, Perth, Western Australia, Australia (A. Martin);; The Canberra Hospital, Canberra, Australian Capital Territory, Australia (T. McDonald, L. Teoh);; Monash Institute of Medical Research, Melbourne, Victoria, Australia (G.M. Nixon);; Alice Springs Hospital, Alice Springs, Northern Territory, Australia (R. Roseby);; Royal Children’s Hospital, Melbourne (S. Ranganathan, M. Soto-Martinez);; Children’s Hospital at Westmead (H. Selvadurai);; Women’s and Children’s Hospital, Adelaide, South Australia, Australia (G. Smith);; Mater Children’s Hospital, Brisbane, Queensland, Australia (S. Suresh);; Royal Children’s Hospital, Brisbane (C.E. Wainwright)

**Keywords:** Streptococcus pneumoniae, empyema, child, bacteria, polymerase chain reaction, Australia, PCR, PCV7, vaccines, 7-valent pneumococcal conjugate vaccine, serotypes, research

## Abstract

An increase in the incidence of empyema worldwide could be related to invasive pneumococcal disease caused by emergent nonvaccine replacement serotypes. To determine bacterial pathogens and pneumococcal serotypes that cause empyema in children in Australia, we conducted a 2-year study of 174 children with empyema. Blood and pleural fluid samples were cultured, and pleural fluid was tested by PCR. Thirty-two (21.0%) of 152 blood and 53 (33.1%) of 160 pleural fluid cultures were positive for bacteria; *Streptococcus pneumoniae* was the most common organism identified. PCR identified *S. pneumoniae* in 74 (51.7%) and other bacteria in 19 (13.1%) of 145 pleural fluid specimens. Of 53 samples in which *S. pneumoniae* serotypes were identified, 2 (3.8%) had vaccine-related and 51 (96.2%) had nonvaccine serotypes; 19A (n = 20; 36.4%), 3 (n = 18; 32.7%), and 1 (n = 8; 14.5%) were the most common. High proportions of nonvaccine serotypes suggest the need to broaden vaccine coverage.

## Abstract 2

Most infections were caused by non–7-valent pneumococcal conjugate vaccine serotypes.

Empyema in children is a relatively uncommon disease that occurs in 0.7% of children with pneumonia (*1*). Many organisms cause empyema in children; *Streptococcus pneumoniae* is the most common (*2**–**6*). Other important causes, which are becoming increasingly frequent in several countries, are methicillin-sensitive *Staphylococcus aureus* (MSSA) (*2**,**7**,**8*) and methicillin-resistant *S. aureus* (MRSA). The latter is particularly problematic in indigenous communities (*9*). Other commonly identified organisms include *S. pyogenes, Haemophilus influenzae*, *Mycoplasma pneumoniae*, *Pseudomonas aeruginosa*, and other *Streptococcus* spp (*10*). The identification of causative organisms is usually determined by standard blood or pleural fluid cultures. Cultures are limited in that the yield can be as low as 8% (*11*), possibly because of prior antimicrobial drug treatment. Molecular techniques, such as PCR, are more sensitive in detecting causative organisms than standard culture (*11*) but are not routinely employed in laboratories for clinical use.

The 7-valent pneumococcal conjugate vaccine (PCV7) (Prevenar; Wyeth, Philadelphia, PA, USA) was introduced in Australia for immunocompromised and indigenous children <2 years of age in 2001 and was added to the national immunization schedule for all children <2 years in 2005 (www.medicareaustralia.gov.au/public/services/acir/index.jsp). Of >90 pneumococcal serotypes, the 7 included in the vaccine were responsible for 50%–70% of invasive pneumococcal disease (IPD) in children in most populations at the time of its development (*12*).

Many reports from around the world suggest an increase in the incidence of empyema in children (*1**,**6**,**13**–**19*). The reasons for this increase are unknown but may be related to IPD caused by emergent nonvaccine replacement serotypes, particularly serotypes 1, 3, and 19A after the introduction of PCV7 (*14**–**20*). However, this theory is controversial because several studies identified an increase in empyema prevalence before the introduction of PCV7 (*5**,**21**,**22*). Because no Australian data exist on the bacterial causes of empyema, it is difficult to determine whether incidence in Australia is similar to reported trends in North America (*14**,**22*), the United Kingdom (*5**,**6**,**16**,**18**,**21*), Spain (*15**,**17*), and France (*13*).

The aims of this study were to identify the bacterial causes of empyema in children by using molecular techniques and to assess the efficacy of PCV7 by using molecular typing of invasive pneumococcal disease serotypes. This information may be helpful in deciding which of the newer conjugate pneumococcal vaccines should be introduced into national vaccination programs.

## Methods

The Australian Research Network in Empyema was established in April 2007 and comprises all 13 major tertiary pediatric hospitals from all states and territories. Children with empyema were prospectively recruited over a 2-year period until April 2009.

### Patients

A case of childhood empyema was defined by the principal site investigators as the presence of pus cells in the pleural fluid or bacteria isolated from the pleural fluid of a child with fever, respiratory symptoms, raised serologic inflammatory markers, and pleural fluid present on ultrasound image, chest radiograph, or computed tomography scan. Children with postoperative effusions were excluded.

Clinical data collected included age, sex, indigenous status, area of residence, risk factors, congenital or chromosomal abnormality, anatomic or functional asplenia, immunocompromise, and chronic illness. Vaccination status of recruited patients was obtained by either review of the child’s hand-held health records (Blue Book), contacting the Australian Childhood Immunisation Registry (with parental permission) for patients <7 years of age (www.medicareaustralia.gov.au/public/services/acir/index.jsp), or, if these validated sources were unavailable, parental recall.

### Microbiologic Investigations

Blood and pleural fluid specimens were cultured at local hospital microbiology laboratories by standard culture method. If growth was detected, Gram staining was performed and liquid media were subcultured onto horse blood agar. Isolates were identified by using conventional methods. Colonies resembling *S. pneumoniae* that contained gram-positive diplococci were identified by optochin- susceptibility and bile-solubility testing.

A separate aliquot of pleural fluid (in ideal circumstances, 10 mL) was collected, labeled according to the central coordinator’s de-identification and specimen-tracking database, and stored at −20°C. Pleural fluid specimens were transported in batches on dry ice by a commercial transport company to the Centre for Infectious Disease and Microbiology Laboratory, Westmead Hospital, Westmead, New South Wales, Australia, for processing.

### *Streptococcus pneumoniae* PCR

Total nucleic acid was extracted from pleural fluid specimens by using either NucliSENS easyMAG Total Nucleic Acid Extractor (bioMérieux Australia Pty Ltd, Sydney, NSW, Australia) with enzymes and lysis buffer provided, or SIGMA GenElute Mammalian Genomic DNA Miniprep Kit (Sigma-Aldrich, Sydney, NSW, Australia) with lysis buffer provided plus proteinase K, following the manufacturer’s instructions.

*S. pneumoniae* PCR targeting the autolysin gene (*lytA*) was performed by using a TaqMan probe and primers as described by McAvin et al. (*23*), except that the result was read by spectrofluorometry and interpreted as described by Poddar et al. (*24*). Briefly, a 25-µL PCR containing 1.5 mmol/L MgCl_2_, 200 µmol/L dNTPs, 200 nmol/L of each primer, 120 nmol/L probe, 1.23U HotStar*Taq* DNA polymerase, and 10 µL of total nucleic acid yielded a 101-bp product. The PCR cycling conditions included initial denaturation at 95°C for 15 min, followed by 45 cycles at 96°C for 10 s, 63°C for 1 min, and a final extension step of 72°C for 2 min. The endpoint results were analyzed by calculating the postread to preread ratio. Samples with ratios of >2.78 were reported as positive and confirmed by using pulsed-field gel electrophoresis on a 2% gel, at 200 V for 40 min. Samples with ratios of <1.21 were reported as negative. The limit of detection of the assay was 6 CFU/mL.

### Pneumococcal Serotype Identification

All samples in which *S. pneumoniae* was detected by PCR were examined by multiplex PCR reverse line blot (mPCR/reverse line blot [RLB]) to identify serotypes individually or in small groups of related serotypes (*25**,**26*). If serogroup 6 was identified, serotype-specific PCRs targeting *wciN* (to distinguish serotypes 6A and 6C) and the *wciP* single-nucleotide polymorphism, which distinguishes serotypes 6A and 6C from 6B (*27*), were performed. Samples that gave no signals in mPCR/RLB (result recorded as below detection level) and those in which only the *S. pneumonia*e positive control probes targeting *ply* or *lytA* produced signals (nontypeable) were further tested when sufficient DNA remained, by PCR and sequencing of the *cpsA-B* region of the capsular polysaccharide synthesis (*cps*) gene cluster, as described and validated (*28*).

### PCR for Other Pathogens

All pleural specimens were tested by separate in-house PCRs for the presence of *Haemophilus influenzae, Mycoplasma pneumoniae*, *Chlamydia pneumoniae*, and *Staphylococcus aureus* DNA*.* *M. pneumoniae* and *C. pneumoniae* PCR used fluorogenic probes with endpoint analysis in a fluorometer. Primers and probes were as follows: *C. pneumoniae* (target PstI fragment): primers Lab2f, 5′-GG AGA TAA AAT GGC TGG ACG-3′; Lab 2r, 5′-TAT GGC ATA TCC GCT TCG G-3′; probe Lab2p, 5′-6-FAM CAC GGA AAT AAA GGT GTT GTT TCC AAA ATCG-6-TAMRA-3′ (*29*); *M. pneumoniae* (target P1 protein gene): primers MYP-Fw, 5′-TCA GGT CAA TCT GGC GTG-3′; MYP-Rv, 5′-TCA AAC AGA TCG GCG ACT G-3′; probe MYP, 5′-(6-FAM) AGT TAC CAA GCA CGA GTG ACG GAA A-3′ (BHQ-1).

Conventional agarose gel electrophoresis was used for *H. influenzae* PCR. Primers used were Hinf OmP 6F, 5′-AAT GGT GCT GCT CAA ACT TT-3′; and Hinf Omp 6R, 5′-TCT AAG ATT TGA ACG TAT TCA CC-3′.

Testing for *S. aureus* DNA was undertaken by using a commercial multiplex-tandem PCR targeting the *S. aureus* nuclease gene *nuc,* and methicillin-resistance gene *mecA* (MRSA Easy-plex assay kit; AusDiagnostics, Sydney, New South Wales, Australia), as recommended by the manufacturer.

### Assessment of Data Accuracy

To assess the completeness of case ascertainment, we contacted the coding departments of all participating hospitals at the end of the study and asked them to provide data on the number of children 0–18 years of age with empyema (classified according to International Classification of Diseases [ICD] codes J86.0 [pyothorax with fistula] or J86.9 [pyothorax without fistula]) who were discharged from each hospital from April 1, 2008, through April 30, 2009. This period was chosen because it represented a time when all hospitals were actively recruiting. These data were compared with our own.

Descriptive statistics were used for all analyses. No power calculation was required because this was an epidemiologic study aiming to capture all cases of empyema.

This study was approved by the local human research ethics committee at each site, and registered with The Australian and New Zealand Clinical Trial Registry (ACTRN12607000476437). Informed parental consent was obtained for each patient before blood and pleural fluid samples were collected.

## Results

A total of 174 children were recruited over a 2-year period from April 2007 through April 2009, with a median recruitment rate of 6 (range 0–19) per month. Over a 1-year period, study sites recruited a median of 51.5% (range 0%–200%) of actual admissions identified by ICD coding discharge summaries. Demographic information was available from 172 patients (Table 1); of these, 70 (40.7%) were fully vaccinated (median age 2.1 years, range 0.4–5.3 years); 18 (10.5%) were partially vaccinated (median age 4.2 years, range 0.6–5.4 years); 56 (32.6%) were not vaccinated (median age 7.7 years, range 1–15.5 years); and 28 (16.3%) had no record of vaccination status (median age 6.1 years, range 0.6–14.4 years).

**Table 1:** Characteristics of 172 children with empyema, Australia, 2007–2009*

Characteristic	Value
Median age, y (range)	3.9 (0.4–15.5)
Sex	
M	93 (54.1)
F	79 (45.9)
Indigenous status	
Aboriginal	8 (4.7)
Other	160 (93.0)
Not recorded	4 (2.3)
State/territory	
Queensland	59 (34.3)
New South Wales	52 (30.2)
Victoria	37 (21.5)
Western Australia	16 (9.3)
South Australia	4 (2.3)
Tasmania	2 (1.2)
Australian Capital Territory	2 (1.2)
Northern Territory	0
Chronic respiratory diseases	7 (4.0)
Congenital diseases	3 (1.7)
Potentially immunocompromised	6 (3.5)
Cardiac disease	1 (0.6)

*Values are no. (%) unless otherwise indicated.

Of the 174 children recruited, culture results were available for 172; 140 had blood and pleural fluid cultured, 20 had only pleural fluid cultured, and 12 had only blood cultured. Of 152 blood and 160 pleural fluid cultures, 120 (78.9%) and 107 (66.9%), respectively, were negative. The bacteria isolated are shown in Table 2.

**Table 2:** Bacteria isolated by culture of blood and pleural fluid samples and by PCR of pleural fluid samples from children with empyema, Australia, 2007–2009*

Organism	No. (%) positive samples		
	Blood culture, n = 152	Pleural fluid	
		Culture, n = 160	PCR, n = 145
*Streptococcus pneumoniae*	19 (12.5)	12 (7.5)	74 (51)
*S. pyogenes*	3 (2.0)	14 (8.8)	NA
*S. milleri*	NA	4 (2.5)	NA
MSSA	1 (0.7)	11 (6.8)	6 (4.1)
MRSA	1 (0.7)	6 (3.8)	7 (4.8)
Coagulase-negative staphylococci	4 (2.6)	2 (1.3)	NA
*Haemophilus influenzae*	1 (0.7)	NA	4 (2.8)
*Mycobacterium tuberculosis*	NA	1 (0.6	NA
*Pseudomonas aeruginosa*	NA	1 (0.6)	NA
*Mycoplasma pneumoniae*	NA	NA	1 (0.7)
*Chlamydia pneumoniae*	NA	NA	1 (0.7)
Other†	4 (2.6)	4 (2.5)	NA

*NA, not applicable; MSSA, methicillin-susceptible *Staphylococcus aureus*; MRSA, methicillin-resistant *S.s aureus*. †Blood cultures: 1 isolate each of *Streptococcus sanguinis; Staphylococcus hominis* (from a specimen in which *S. aureus* was also isolated); *Neisseria meningitidis*; and *Actinomyces naeslundii.* Pleural fluid: 1 isolate each of *Streptococcus oralis; Staphylococcus cohni; Eikenella corrodens;* and *Bacteroides fragilis* (the last 2 from the same specimen, from which *S. milleri* was also isolated*).* Both MRSA and MSSA were cultured from 1 pleural fluid specimen.

**Figure 1:**
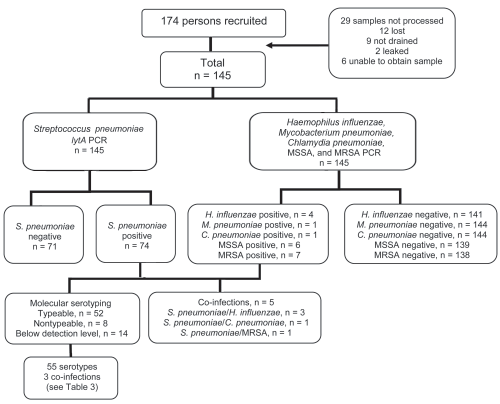
Flow diagram for PCR testing for bacterial pathogens in samples from children with empyema, Australia, 2007–2009. MSSA, methicillin-resistant *Staphylococcus aureus*; MRSA, methicillin-resistant *S. aureus*.

PCRs for *S. pneumoniae*, *H. influenzae*, *M. pneumoniae*, *C. pneumoniae*, and *S.* *aureus* were performed on 145 (83.3%) pleural fluid specimens (Figure 1). One or more of these organisms was detected by PCR in 88 specimens: *S. pneumoniae,* 74 (51.0%); *S. aureus*, 13 (9.0%; 1 with *S. pneumoniae*); *H. influenzae*, 4 (2.8%; 3 with *S. pneumoniae*); *M. pneumoniae*, 1 (0.7%); *C. pneumoniae*, 1 (0.7%; with *S. pneumoniae*). Of the 13 *S.* *aureus*–positive specimens, 6 were MSSA and 7 MRSA.

Pneumococcal serotypes were identified in 52 (70.3%) of 74 *S. pneumoniae* PCR-positive specimens; 3 specimens contained 2 serotypes (Table 3). Sufficient DNA could not be obtained for 22 *S. pneumoniae–*positive specimens to identify serotypes, including 14 in which there was no signal in mPCR/RLB and 8 in which 1 or both *S. pneumoniae*–specific (*ply* and/or *lytA*) probe signals, but none of the serotype-specific probes, were positive on RLB.

**Table 3:** *Streptococcus pneumoniae* serotypes identified in 52 PCR-positive specimens from children with empyema, Australia, 2007–2009*

Serotype	No. (%) specimens
PCV7 serotypes	2
14	1 (1.8)
9V/9A	1 (1.8)
Nonvaccine serotypes	53
19A	20 (36.4)
3	18 (32.7)
1	8 (14.5)
7F/7A	2 (3.6)
22F/22A	2 (3.6)
6C	1(1.8)
15F	1 (1.8)
21	1 (1.8)

*Two serotypes were identified in 3 specimens: 19A/3, 19A/1, and 6C/15F. The multiplex PCR reverse line blot assay cannot distinguish some pairs or groups of closely related serotypes, including 7F/7A, 22F/22A, and serogroup 6. Individual serotypes within serogroup 6 were distinguished by using serotype-specific PCR. PCV-7, 7-valent pneumococcal conjugate vaccine.

**Figure 2:**
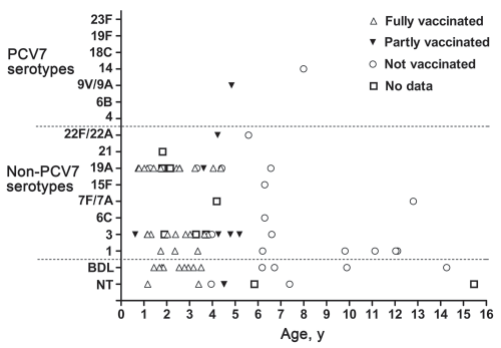
*Streptococcus pneumoniae* serotype distribution in relation to age and vaccination status of children with empyema, Australia, 2007–2009. PCV7, 7-valent pneumococcal conjugate vaccine.

Vaccination status was available for 45 (86.5%) of 52 children who had a pneumococcal serotype detected on PCR. The effect of vaccination status on the acquisition of specific serotypes, in relation to age and vaccination status, was assessed (Figure 2).

## Discussion

This study supports previous reports from different countries that have identified *S. pneumoniae*, *S. aureus,* and *S. pyogenes* as notable causes of childhood empyema (*2**–**4**,**8**,**17**,**21*). Most (96.4%) of identified pneumococcal serotypes were nonvaccine related, reflecting the effectiveness of the PCV7. Furthermore, this study highlights that PCR is more sensitive than culture for identifying pathogens.

Data on bacterial causes of childhood pneumonia in this geographic region are lacking (*30*). Before this study, the most comprehensive data relating to pneumococcal serotypes causing disease in children were from routine typing of sterile site isolates from cases of IPD reported to the Nationally Notifiable Diseases Surveillance System. However, surveillance of IPD does not specifically report on empyema.

By far, the most common organism identified was *S. pneumoniae*. A variety of organisms other than *S. pneumoniae* were detected by culture and PCR. After *S. pneumoniae*, *S. aureus* was the next most common pathogen, which was identified by culture in 17 pleural fluid and 2 blood specimens (12 MSSA, 7 MRSA) and by PCR in 13 specimens (Table 2). The occurrence of MRSA as a cause of community-acquired pneumonia and empyema is of particular concern because it is associated with more severe disease and a higher rate of complications than MSSA (*9*).

*H. influenzae* was detected by PCR in 4 children, 3 of whom also had positive PCR results for *S. pneumoniae*. *S. pyogenes*, *Mycobacterium tuberculosis,* and *P. aeruginosa* were isolated only by culture (individual PCR assays for these organisms were not available), and *M. pneumoniae* was detected by PCR in 1 child (Figure 1). All of these organisms are recognized causes of empyema in children (*10*). Although *C. pneumoniae* is a recognized cause of lower respiratory tract infection in children (*31**,**32*), its contribution to empyema has not been investigated previously. It was detected by PCR in only 1 child, with *S. pneumoniae* in the same specimen, which suggests that *C. pneumoniae* is not a major cause of empyema in children.

This study confirmed the findings of others, demonstrating enhanced sensitivity of molecular techniques (*11**,**33**–**35*). PCR detected many more *S. pneumoniae* isolates in pleural fluid than in cultures (51.0% vs. 7.5%), and thus routine use of PCR-based serotype identification in children with empyema may improve the accuracy of pneumococcal disease surveillance, which is essential for development of new vaccines with broader range of pneumococcal serotypes. In contrast, however, for 4 patients who had a culture positive for *S. aureus*, PCR results were negative. We are unclear why this occurred, but this PCR was performed last in the sequence, and results may have been due to insufficient DNA. Although PCR did not increase the yield of *S. aureus*, it can detect it more rapidly than culture, enabling rapid change to appropriate therapy, especially when MRSA is found*.*

Reports on childhood empyema pneumococcal serotype distribution from Europe and the United States show differences. Studies in Spain (*8**,**17*), the United Kingdom (*16**,**21**,**36*), and the United States (*14*) have reported a predominance of serotype 1, while in other US studies, 19A is the most common serotype (*20**,**22*); both are non–PCV7 serotypes. Bekri et al. (*13*) identified serotypes 1 and 19A as emerging serotypes in France and also showed that serotype 1 was predominant in children >5 years of age; serotype 19A appeared to only affect children <5 years of age. This age distribution was similarly reported in another study (*37*). The serotype distribution in our study was similar; serotypes 1, 3, and 19A were predominant (Figure 2), and most serotypes 3 and 19A were identified in children <5 years of age, similar to results previously reported (*37*). Although serotype 1 infections were identified across all age groups, most were in children >5 years of age.

We were reassured that only 2 children in our study had serotypes covered by PCV7; 1 child was covered partially, and the other had not been vaccinated. Overall, this study suggests the efficacy of the PCV7, as previously confirmed (*38*). However, public health authorities should be concerned that most pneumococcal infections were caused by nonvaccine serotypes, possibly related to replacement disease after the introduction of PCV7 onto the national vaccination schedule in 2005. We do not have serotype data specific to empyema prior to 2005, but this has occurred in other countries and affects all IPD, including meningitis (*39*). Most studies that compare pre– and post–pneumococcal vaccine effects have shown near extinction of PCV7 serotypes, along with dramatic increases in nonvaccine serotypes, predominantly 1, 3, and 19A (*14**,**15**,**17**,**20*).

Although the reasons behind serotype changes have not been determined fully, ongoing enhanced surveillance may help clarify them over time, enabling us to predict future serotype trends and tailor new vaccines accordingly. This ability is particularly relevant as 2 new vaccines with broader coverage of pneumococcal serotypes—10-valent pneumococcal conjugate vaccine, with additional serotypes 1, 5, and 7F, and 13-valent pneumococcal conjugate vaccine, with additional serotypes 1, 3, 5, 6A, 19A, and 7F—are being are being added to national vaccination schedules. The 10-valent vaccine offers protection against nontypeable *H. influenzae* through the use of an *H. influenzae* conjugate protein.

Our study has several limitations, nevertheless. First, we cannot know whether the number of children recruited in this study is an accurate snapshot of Australia’s true empyema prevalence in children. However, after comparing ICD empyema codes with study recruitment rates over a 1-year period, we determined that we recruited a median of 51.5% (range 0%–200%) of empyema patients admitted to all major pediatric tertiary hospitals recorded by ICD. A limitation of this approach is that we were not able to verify the coding accuracy in each of the 13 hospitals. One likely reason why we did not capture all the cases may be because some patients received treatment from physicians at participating centers who were unaware of the study. Also, some children may have received treatment at smaller rural hospitals where the study was not conducted, even though we have recently shown that most patients are treated in 1 of the tertiary pediatric hospitals included in this study (*1*). We recruited patients from all states and territories in Australia, which is the strength of the study. A limitation of the PCR data is that the bacteria assessed were restricted to *H. influenzae, M. pneumoniae*, *C. pneumoniae,* and *S. aureu*s. They are all potentially important bacterial pathogens in empyema in children, however (*10*). The use of broader PCR, such as 16sPCR, may have detected more organisms, but it is an expensive test and our previous experience suggests that the yield of notable pathogens is poor and that positive results often reflect contamination.

In conclusion, we have demonstrated a wide variety of bacterial causes for empyema in children. Most infections were caused by non–PCV7 pneumococcal serotypes, which suggests that coverage of pneumococcal serotypes by vaccines needs to be broadened. Ongoing enhanced molecular surveillance is required, particularly to assess the effects of newer vaccines, such as 10-valent and 13-valent pneumococcal conjugate vaccines.
